# Caffeine and a selective adenosine A_2A _receptor antagonist induce sensitization and cross-sensitization behavior associated with increased striatal dopamine in mice

**DOI:** 10.1186/1423-0127-17-4

**Published:** 2010-01-15

**Authors:** Chih W Hsu, Chin S Wang, Ted H Chiu

**Affiliations:** 1 Department of Emergency Medicine, Tzu Chi General Hospital, Taiwan; 2 Institute of Medical Sciences, Tzu Chi University, Hualien, 970, Taiwan; 3 Institute of Pharmacology and Toxicology, Tzu Chi University, Hualien, 970, Taiwan; 4 School of Medicine, Tzu Chi University, Hualien, 970, Taiwan; 5 Department of Pharmacology, Tzu Chi University, Hualien, 970, Taiwan

## Abstract

**Background:**

Caffeine, a nonselective adenosine A_1 _and A_2A _receptor antagonist, is the most widely used psychoactive substance in the world. Evidence demonstrates that caffeine and selective adenosine A_2A _antagonists interact with the neuronal systems involved in drug reinforcement, locomotor sensitization, and therapeutic effect in Parkinson's disease (PD). Evidence also indicates that low doses of caffeine and a selective adenosine A_2A _antagonist SCH58261 elicit locomotor stimulation whereas high doses of these drugs exert locomotor inhibition. Since these behavioral and therapeutic effects are mediated by the mesolimbic and nigrostriatal dopaminergic pathways which project to the striatum, we hypothesize that low doses of caffeine and SCH58261 may modulate the functions of dopaminergic neurons in the striatum.

**Methods:**

In this study, we evaluated the neuroadaptations in the striatum by using reverse-phase high performance liquid chromatography (HPLC) to quantitate the concentrations of striatal dopamine and its metabolites, dihydroxylphenylacetic acid (DOPAC) and homovanilic acid (HVA), and using immunoblotting to measure the level of phosphorylation of tyrosine hydroxylase (TH) at Ser31, following chronic caffeine and SCH58261 sensitization in mice. Moreover, to validate further that the behavior sensitization of caffeine is through antagonism at the adenosine A_2A _receptor, we also evaluate whether chronic pretreatment with a selective adenosine A_2A _antagonist SCH58261 or a selective adenosine A_1 _antagonist DPCPX can sensitize the locomotor stimulating effects of caffeine.

**Results:**

Chronic treatments with low dose caffeine (10 mg/kg) or SCH58261 (2 mg/kg) increased the concentrations of dopamine, DOPAC and HVA, concomitant with increased TH phosphorylation at Ser31 and consequently enhanced TH activity in the striatal tissues in both caffeine- and SCH58261-sensitized mice. In addition, chronic caffeine or SCH58261 administration induced locomotor sensitization, and locomotor cross-sensitization to caffeine was observed following chronic treatment of mice with SCH58261 but not with DPCPX.

**Conclusions:**

Our study demonstrated that low dosages of caffeine and a selective adenosine A_2A _antagonist SCH58261 elicited locomotor sensitization and cross-sensitization, which were associated with elevated dopamine concentration and TH phosphorylation at Ser31 in the striatum. Blockade of adenosine A_2A _receptor may play an important role in the striatal neuroadaptations observed in the caffeine-sensitized and SCH58261-sensitized mice.

## Background

Caffeine, a nonselective adenosine A_1 _and A_2A _receptor antagonist, is the most widely used psychoactive substance in the world. In spite of debate about the abuse potential of caffeine, a literature review of human caffeine withdrawal has provided sufficient evidence to warrant the inclusion of caffeine withdrawal as a chemical dependent disorder [1]. In animal models, caffeine causes motor sensitization [2-4], conditioned place preference [4-6], and cross-sensitization to locomotion elicited by nicotine and amphetamine [2,7]. Furthermore, our previous study [4] has demonstrated that caffeine and SCH58261, a selective adenosine A_2A _receptor antagonist, but not a selective A_1 _adenosine receptor antagonist DPCPX, can induce reward and behavioral sensitization.

Evidence indicates that mesolimbic dopaminergic pathway mediates the reinforcement and behavioral sensitization of caffeine. Many studies also suggest that caffeine interacts with the nigrostriatal dopaminergic pathway to modulate its motor-stimulating effect. The anatomical and functional interactions between the adenosine and dopamine receptors in the striatum have been recently reviewed [8-10].

Interestingly, two large prospective epidemiological studies have linked coffee drinking to a reduced risk of developing Parkinson's disease (PD) [11,12]. There is also evidence to indicate that administration of caffeine and adenosine A_2A _antagonists have therapeutic effects in animal models of PD [13,14]. Many studies have demonstrated that A_2A _antagonists attenuated the 1-methyl 4-phenyl 1,2,3,6-tetrahydropyridine (MPTP)-induced neurodegeneration [15] and enhanced the therapeutic effect of various dopamine agonists, including L-DOPA in animals [15-18]. Kelsey et al. [14] found that caffeine and a selective adenosine A_2A _antagonist SCH58261, but not a selective adenosine A_1 _agonist N^6^-cyclopentyladenosine and a selective A_2A _antagonist 8-cyclopentyltheophylline, exhibited both monotherapeutic and adjunctive therapeutic effects in an established model of PD. These observations indicate that caffeine has neuroprotective effect on nigrostriatal dopaminergic pathway via antagonism of adenosine A_2A _receptors.

Drug reward and voluntary motor movement are the two main functions of the dopamine system. Thus, dopamine modulation is central to the disorders of drug addiction and PD. The striatum is the main receiving area of the basal ganglia, and about 95% of the efferent striatal neurons consist of GABAergic medium spiny neurons. These neurons receive a modulatory input from midbrain dopaminergic neurons. The ventral striatum, comprised of the nucleus accumbens, receives its dopaminergic input from the ventral tegmental area and this projection constitutes the mesolimbic pathway, which is involved in drug reinforcement, addiction, and behavioral sensitization [19]. The dorsal striatum, comprised of the caudate-putamen, receives its dopaminergic input from the substantia nigra pars compacta and this projection constitutes the nigrostriatal pathway, which is involved in PD.

Since caffeine and selective A_2A _antagonists induce the reinforcement and sensitization behaviors, and exhibit the therapeutic effects in animal models of PD, which are mediated by mesolimic and nigrostrial dopaminergic pathways projected to the striatum, it is reasonable to hypothesize that caffeine and selective A_2A _antagonists can modulate the neuroadaptation of dopaminergic neurons in the striatum. Indeed, the expression of adenosine A_2A _receptors in the brain is mostly limited to the striatum [20]. Dopamine depletion or blockade of dopamine receptors significantly impairs the motor and discriminative stimulus effects of caffeine [21]. Chronic high dosages (25 and 50 mg/kg/day, twice daily) but not low dosage (10 mg/kg/day, twice daily) of caffeine were associated with elevated levels of dopamine and 5-hydroxytriptamine but decreased level of dihydroxyphenylacetic acid (DOPAC) in the rat striatum [22]. Increased expression of tyrosine hydroxylase mRNA was found in the ventral tegmental area and substantia nigra pars compacta of chronic caffeine-treated (20-80 mg/kg × 9 days) rats [23].

Sensitization of locomotor activity and conditioned place preference are the most commonly studied paradigms, which reflect the incentive motivational properties of drugs believed to contribute to the intensification of drug craving and compulsive drug-seeking behavior [24]. Our previous and other studies have demonstrated that 15 and 20 mg/kg of caffeine induced the sensitization of locomotor activity [2-4], but conditioned place preference was observed only with less than 10 mg/kg caffeine [4-6]. It has been found that the psychomotor stimulant effect of low doses of caffeine is mediated by the inhibition of adenosine A_2A _receptors, involving dopamine-dependent as well as dopamine-independent mechanisms, whereas higher doses of caffeine elicit locomotor depression, most likely acting through antagonism at adenosine A_1 _receptors [8]. To investigate whether caffeine and A_2A _antagonists can modulate the dopaminergic system in the striatum that underlies drug addiction and treatment of PD, we chose low dosage of caffeine (10 mg/kg/day) and A_2A _antagonist SCH58261 (2 mg/kg/day), which can induce the sensitization of locomotor activity and reward behavior, to evaluate the roles of dopaminergic neurons in the striatum. To further substantiate that the behavioral sensitization effect of caffeine is mediated by the antagonism of adenosine A_2A _receptor, we also assessed whether chronic pretreatment with a selective adenosine A_2A _antagonist SCH58261 can potentiate the behavioral effects of caffeine. Our results indicate that following chronic administration with low dosages of caffeine or SCH58261, a time-dependent locomotor sensitization was found. In addition, cross-sensitization to caffeine was observed after chronic treatment with SCH58261 but not DPCPX, a selective adenosine A_1 _receptor antagonist. The striatal contents of DA, its metabolites, DOPAC and HVA (homovanilic acid), were elevated after same dosages of chronic caffeine and SCH58261 administration. The elevation of DA and its metabolites were associated with the enhanced phosphorylation of tyrosine hydroxylase at Ser31, the active form and rate-limiting enzyme in catecholamine biosynthesis. These data indicate that striatal dopaminergic pathways play an important role in mediating the locomotor sensitization and reward effects after chronic administration with caffeine and selective adenosine A_2A _antagonist SCH58261.

## Materials and methods

### Animals

Male C57BL/6 mice, purchased from the National Laboratory Animal Breeding and Research Center (Taipei, Taiwan), were established at the Laboratory Animal Center, Tzu Chi University. Mice weighing 25-35g were used in the present study. All experimental procedures were carried out in accordance with the guidelines of the Institutional Animal Care and Use Committee of Tzu Chi University. Every effort was made to minimize the suffering and the number of animals used.

### Drugs

Caffeine, DPCPX (8-cyclopentyl-1,3-dipropylxanthine) and SCH-58261 (5-amino-7-(β-phenylethyl)-2-(8-furyl) pyrazolol [4,3-e] - 1,2,4 - triazolol [1,5-c] pyrimidine) were purchased from Sigma-RBI (Taipei, Taiwan). Caffeine was dissolved in saline whereas SCH 58261 and DPCPX were dissolved in dimethyl sulfoxide (DMSO). All drugs were administered i.p. with the dosages specified in each experiment.

### Evaluation of locomotor activity

Locomotor activity was monitored as described previously (Hsu et al., 2009). Briefly, a 2-hr habituation period was routinely used prior to the administration of test drugs. Images of the locomotor activity (distance traveled) were captured by a video camera and the recorded images were transferred to the interface of a computer for processing. The track data stored in a special format were retrieved and analyzed by TrackMot software (Diagnostic & Research Instruments Co., Taoyuan, Taiwan). The activity was summated consecutively for three 10-min intervals following the drug administration. In addition, the total distance traveled for the initial 30 min was also summated for analysis. All animals were used only once.

### Locomotor sensitizing effects following chronic caffeine and SCH 58261 administrations

According to our previous study (Hsu et al., 2009), dosages of caffeine (10 mg/kg) and SCH58261 (2 mg/kg), which induced conditioned place preference, were used in the chronic sensitization experiments. Mice were administered i.p. caffeine or SCH58261 for 5 consecutive days, and after one-day washout, the locomotor activity was monitored for 30 min by administering an acute dose of caffeine (10 mg/kg) or SCH58216 (2 mg/kg) on day 7. Mice were kept on the same dosages of caffeine or SCH58261 for another 4 consecutive days followed by 3-day washout. The locomotor activity on day 15 elicited by an acute dosage of caffeine (10 mg/kg) or SCH58261 (2 mg/kg) was recorded for 30 min. Animals in the control groups received either saline or DMSO. Acute motor stimulating effects of caffeine (10 mg/kg) or SCH58261 (2 mg/kg) on day 1, 7 and 15 were also recorded in the caffeine-treated groups or SCH58261-treated groups for comparison.

### Cross-sensitization effect of caffeine on chronic SCH58261- and DPCPX-treated mice

Mice were administered SCH 58261 (2 mg/kg, i.p.), DPCPX (3 mg/kg, i.p.) or DMSO daily for 14 days. Three days after the last scheduled administration, the locomotor activity of an acute dosage of caffeine (10 mg/kg, i.p.) was recorded for 30 min following 2 hrs habituation. The locomotor activities produced by an acute dosage of caffeine (10 mg/kg, i.p.) between SCH 58261-treated and DMSO-treated groups and between DPCPX-treated and DMSO-treated groups after washout period were compared to assess the locomotor cross-sensitization effect.

### Measurement of dopamine concentration in the striatum of sensitized-mice

Following 3-day washout, mice chronically treated with caffeine, SCH58261, or vehicles were sacrificed by decapitation 30 min after an acute corresponding dosage of caffeine (10 mg/kg) or SCH58261 (2 mg/kg). The brains were removed and placed on an ice-cold surface, and the striata were dissected out immediately under a microscope, weighed, and homogenized in the buffer (ice-cold 0.1 M HCl, 0.1 mM sodium metabisulfate). After centrifugation at 12,000 rpm for 10 min, 100 μl of supernatant was removed and further separated using 0.2 μm pore size filter (Millipore, MA, USA) and centrifuged again at 12,000 rpm for 10 min. Dopamine (DA) and its metabolites DOPAC and HVA in the filtrate were quantitated by reverse-phase high performance liquid chromatography (HPLC) with electrochemical detection [25]. Twenty μl of dialysate were subjected to HPLC-ECD detection. The HPLC consists of a microbore reverse phase column (G.L. Sciences inertsil-2, 5-μm ODS, 250 mm × 1.0 mm, I.D., Tokyo, Japan), a CMA-160 On-line injector (CMA/Microdialysis, Stockholm, Sweden), a microbore LC system with a dual potentiostat amperometric detector BAS-4C and the MF-1020 electrode (Bioanalytical Systems, West Lafayette, IN, U.S.A.), and a Beckman I/O 406 interface with Data Analysis Software (Beckman Instrument Inc., Taiwan). The amount of the amines in the filtrate was corrected by the recovery of a known amount of the internal standard (2,3-dihydroxybutyric acid).

### Western blotting

Following 3-day washout, mice chronically treated with caffeine, SCH58261, or vehicles were sacrificed by decapitation 30 min after an acute corresponding dosage of caffeine (10 mg/kg) or SCH58261 (2 mg/kg). The brains were then removed and the striata were dissected under a microscope on an ice-cold surface and homogenized in the lysis buffer (0.5 mM dithiothreitol, 0.2 mM EDTA, 20 mM HEPES, 2.5 mM MgCl_2_, 75 mM NaCl, 0.1 mM Na_3_VO_4_, 50 mM NaF, 0.1% Triton X-100, and a cocktail tablet containing protease inhibitors (Roche, Mannheim, Germany)). After centrifugation at 12,000 rpm for 30 min, the supernatant was removed and stored at -80°C until assayed. Protein concentrations were determined using the Bio-Rad protein assay kit. Eighty micrograms of protein from each sample were subjected to 10% SDS-polyacrylamide gel electrophoresis followed by electrophoretic transfer to polyvinylidene difluoride membranes. The membranes were immunoblotted using primary antibodies for phospho-Ser31-TH (1:500) (Abcam; Cambridge, UK), total TH (1:2000) (Abcam) or actin (1:10000) (BD Biosciences; US) and followed with a horseradish peroxidase-conjugated secondary antibody (Santa Cruz; Santa Cruz, CA). Finally, the protein bands were visualized on the X-ray film using the chemiluminescence detection system (ECL, Amersham, Berkshire, England). The intensity of the band was quantified with a densitometric analysis (GS-800 Calibrated Densitometer, Bio-Rad), and calculated as the optical density × area of band.

### Statistical analysis

The locomotor activity was calculated for every 10-min recording. In addition, total drug-induced locomotor activities for the entire 30 min on day 1, day 7 and day 15 following drug administrations were summated. Data were expressed as mean ± standard error of the mean (SEM). Data were analyzed for statistical significance using the computer program Prism for two-way ANOVA followed by Bonferroni post-test. In addition, mean ± SEM of total locomotor activity (30 min) was calculated and analyzed by Student's t-test. The concentration of dopamine and its metabolites were normalized by internal standard, and the phosphorylation of TH and total TH were normalized by actin in the striatal homogenates. Data were expressed as mean ± SEM and analyzed by Student's t-test. In all cases, p < 0.05 was considered statistically significant.

## Results

### Locomotor sensitization after chronic caffeine or SCH58261 treatment

**Figure 1:**
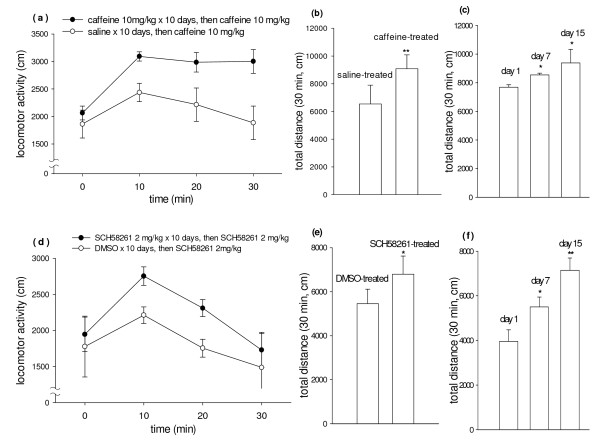
**Locomotor sensitization by repeated caffeine and SCH58261 administration in habituated C57BL/6 mice**. Caffeine (10 mg/kg/day, i.p.), SCH58261 (2 mg/kg/day, i.p.) or vehicles were administered continuously for 10 days except with one washout on day 6. Three days after the last injection of Caffeine, SCH58261 or vehicles, mice were challenged with caffeine (10 mg/kg, i.p.) or SCH58261 (2 mg/kg/day, i.p.) respectively. The horizontal locomotor activity was measured for 30 min. Data represent means ± SEM (n = 4-6). **a, d **The time course of locomotor activity measured over 10-min intervals. **a **caffeine-treated group compared to saline-treated group(P < 0.001) and **d **SCH58261-treated group compared to DMSO-treated group (P = 0.003, by two-way ANOVA); **b, e **Total locomotor activity counts during the 30-min period following acute administration of caffeine or SCH58261. **b **caffeine-treated group compared to saline-treated group (P < 0.01)and **e **SCH58261-treated group compared to DMSO-treated group (P < 0.05); **c, f **Total locomotor activity counts during the 30-min period following acute administration of caffeine in caffeine- treated group and acute administration of SCH58261 in SCH58261-treated group on day1, day7 and day15. **c **locomotor activity of caffeine-treated group on day7 and on day15 compared to locomotor activity of caffeine-treated group on day1, and **f **locomotor activity of SCH58261-treated group on day7 and on day15 compared to locomotor activity of SCH58261-treated group on day1 respectively (*P < 0.05 and **P < 0.01 by Student's test).

The sub-maximal effective dosage of caffeine (10 mg/kg), which induced conditioned place preference (Hsu et al., 2009), was selected to investigate its locomotor sensitization. Mice were given a total of 10 injections with washout on day 6 and day12 to day 14. Three days after the last treatment with caffeine or saline, acute administration of caffeine (10 mg/kg i.p.) caused a greater locomotor response from caffeine- vs vehicle-pretreated mice (Fig. 1a). The result of two-way ANOVA showed F(1,24) = 0.65 and p < 0.001. The total distance traveled for the initial 30 min was increased by 37% following chronic treatment with 10 mg/kg caffeine, significantly different from vehicle control as assessed by Student's t-test (Fig. 1b). In addition, the locomotor activity of acute caffeine administration on day 1, day 7 (after 1-day washout), and day 15 (after 3-day washout) was progressively and significantly enhanced as assessed by Student's t-test (Fig. 1c).

The sub-maximal effective dosage of SCH 58261 (2 mg/kg), which induced conditioned place preference (Hsu et al., 2009), was used to study the locomotor sensitization. The protocol for chronic SCH58261 treatment was analogous to that of caffeine. Three days after the last injection of DMSO or SCH 58261, acute administration of SCH 58261 (2 mg/kg i.p.) resulted in a greater response in the locomotor activity from SCH 58261- as compared with vehicle-pretreated mice. (Fig. 1d). The result of two-way ANOVA showed F(1,18) = 11.74 and p = 0.003. A statistically significant increase of 25% in the total distance traveled for the 30 min duration was also noted following chronic treatment with 2 mg/kg SCH 58261 (Fig. 1e). Further, the locomotor activity of acute SCH58261 administration monitored on day 1, day 7, and day 15 was significantly and progressively enhanced as assessed by Student's t-test (Fig. 1f).

### Cross-sensitization between Caffeine and SCH58261 but not between caffeine and DPCPX

**Figure 2:**
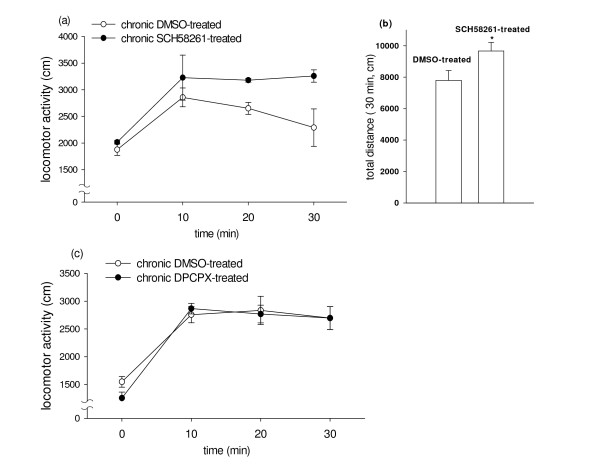
**Cross-sensitization by repeated administration of SCH58261 or DPCPX in habituated C57BL/6 mice**. SCH58261 (2 mg/kg/day, i.p.), DPCPX (3 mg/kg/day, i.p.) or DMSO was administered for 14 days. Three days after the last injection of SCH58261, DPCPX or DMSO, mice were challenged with caffeine (10 mg/kg, i.p.) The horizontal locomotor activity was measured for 30 min. **a **The time course of locomotor activity measured over 10-min intervals. Data represent means ± SEM (n = 3). P < 0.001 versus DMSO-treated group (by two-way ANOVA). **b **Total locomotor activity counts during the 30-min period following acute administration of caffeine. Data represent means ± SEM. P < 0.01 versus DMSO-treated group (by Student's test). **c **The time course of locomotor activity measured over 10-min intervals. Data represent means ± SEM (n = 4). Data showed no significant difference between DPCPX-treated and DMSO-treated group.

After the last injection of SCH 58261 and followed by a 3-day washout period, acute challenge with caffeine (10 mg/kg i.p.) caused a greater response in the locomotor activity from SCH 58261- vs vehicle-pretreated mice. (Fig. 2a). The result of two-way ANOVA showed F(1,12) = 29.07 and p < 0.001. Chronic treatment with 2 mg/kg SCH 58261 also resulted in a 24.5% increase in total distance traveled, with p < 0.01 (Fig. 2b). No significant difference was observed in the locomotor activity from DPCPX- vs vehicle-pretreated mice, challenged with caffeine (Fig. 2c).

### Chronic caffeine and SCH58261 administrations were associated with significant changes in monoamine systems in the striatum

**Figure 3:**
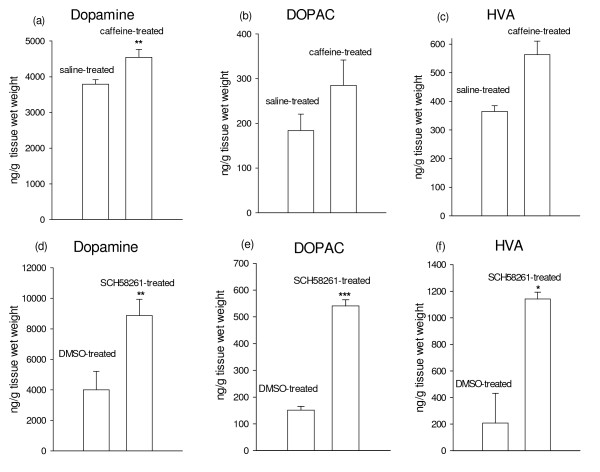
**Effect of chronic caffeine and SCH58261 administration on the striatal amines (n = 3-4)**. **a **DA leves in the striatum after chronic caffeine treatment was increased. Data represent means ± SEM. P < 0.01 versus saline-treated group (by Student's test). **b, c **Increase in DOPAC and HVA levels were also noted but were not statistically significant. **d **DA level in the striatum after chronic SCH58261 treatment was increased. Data represent means ± SEM. P < 0.01 versus DMSO-treated group (by Student's test). **e, f **Increase in DOPAC and HVA levels were also noted. P < 0.001 and P < 0.05 versus DMSO-treated group respectively (by Student's test).

The effect of chronic caffeine and SCH58261 administrations on striatal amines were shown in Fig. 3. Chronic caffeine treatment elevated the striatal DA level by 21% (t = -5.09, P < 0.01). The levels of DOPAC and HVA were increased by 53 and 54%, respectively although they were not statistically significant. Chronic SCH58261 treatment increased the striatal DA content by 119% (t = -4.63, P < 0.01). Similarly, DOPAC and HVA levels in the striatum were also increased by 262% and 456%, respectively, following chronic SCH58261 treatment (t = -10.2, P < 0.001; t = -3.91, P < 0.05 respectively).

### Chronic treatment with caffeine and SCH58261 increased TH phosphorylation at Ser31 in the striatum

**Figure 4:**
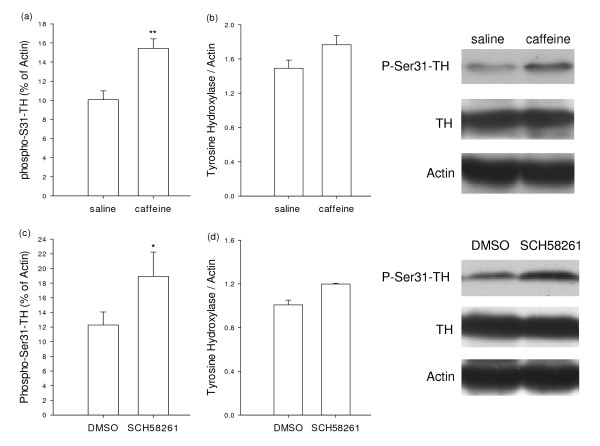
**Representative Western immunoblots of phsopho-S31-TH (a, c) and total TH (b, d)in the striatum of the chronic saline- or caffeine-treated groups and chronic DMSO- or SCH58261-treated groups 30 min after a challenge with caffeine (10 mg/kg) or SCH58261 (2 mg/kg)**. The experiment was repeated at least three times. The *bar graphs *indicate quantitative count of phospho-Ser31-TH and total TH, normalized with actin signals (n = 3-6; *P < 0.05 and **P < 0.01 by Student's test).

Mice were treated with caffeine (10 mg/kg, i.p.) or SCH58261 (2 mg/kg, i.p.) for 10 days as described for the locomotor sensitization experiments. Following 3-day washout period, mice were sacrificed 30 min after acute challenge with caffeine (10 mg/kg) or SCH58261 (2 mg/kg, i.p.). Striatal membrane was prepared for the Western blotting of total TH and phosphor-Ser31-TH expression. As shown in Fig 4, Western blotting demonstrated a statistically significant increase in the proportion of TH phosphorylation at Ser31 after caffeine and SCH58261 treatment (P < 0.01 for caffeine-treated group and P < 0.05 for SCH58261-treated group). A statistically non-significant increase in total TH protein was also observed following chronic caffeine and SCH58261 treatment.

## Discussion

Our previous and other studies have demonstrated that moderate dosages of caffeine (15 and 20 mg/kg) induce locomotor sensitization. However, conditioned place preference was not reported with these dosages of caffeine. Instead, low dosage of caffeine (10 mg/kg), which is more in line with the amount normally ingested in beverages and food, can induce conditioned place preference but the locomotor sensitization has not been reported [2-6,26]. In the present study, we showed that low dosage of caffeine (10 mg/kg) and low dosage of a selective adenosine A_2A _antagonist SCH58261 (2 mg/kg) elicited locomotor sensitization based on the observations that following chronic treatment with the test drugs and allowing for sufficient washout, acute challenge with the test drugs caused a larger response in the drug treated animals when compared to the vehicle-treated ones. Moreover, the expression of the sensitization was progressively enhanced when comparing the motor activity of the same animal on the first, 7th and 15th day following chronic treatment. Chronic treatment with a selective adenosine A_1 _antagonist DPCPX did not demonstrate locomotor sensitization. Our results suggest that chronic administration of low dosages of caffeine or SCH58261, which can induce CPP and behaviour sensitization, are able to elicit neuroadaptive changes similar to those observed with other psychostimulants. The behavioral sensitization of low dose of SCH58261 and the enhancement of acute caffeine-mediated response in SCH58261-sensitized mice strengthen our hypothesis that the effect of caffeine on behavioral reinforcing and sensitization may be mediated through adenosine A_2A _receptor.

Locomotor sensitization, proposed to reflect the increase of the wanting for drug reward, would result from an increase of the responsiveness of dopaminergic neurons to stimuli [24]. Adenosine A_2A _receptors colocalized with dopamine D_2 _receptors in the medium-sized spiny GABAergic neurons are highly and selectively expressed in areas receiving a rich dopamine innervation, i.e., the dorsal and ventral striatum and tuberculum olfactorium [27-29]. Fenu and coworkers [30] have demonstrated that lower dose (10 mg/kg) but not higher dose (25 mg/kg) of caffeine and SCH58261 (3 mg/kg) can cross sensitized to a D_2 _dopamine agonist, bromocriptine. A strong antagonistic interaction between A_2A _and D_2 _receptors in the striatal projection neurons can explain the cross-sensitization between caffeine, or an A_2A _antagonist, and a D_2 _dopamine agonist. Activation of adenosine A_2A _receptors and dopamine D_2 _receptors produce the opposite response of increasing and decreasing the cAMP formation, respectively [31,32]. This results in the opposite regulation of the activity of cAMP-dependent protein kinase involved in modulating the activity of numerous phosphoproteins and transcription factors, which control the expression of immediate early genes, such as c-fos and zif-268, leading to long-term adaptive responses [8,10]. Consequently, antagonism of A_2A _receptors by caffeine and SCH58261 may directly facilitate the actions of D_2 _receptors on striatopallidal neurons. Therefore, it is reasonable to assume that chronic treatment with a selective A_2A _receptor antagonist, analogous to the chronic treatment with caffeine, can result in behavioral sensitization and cross-sensitization.

Our results also showed that chronic treatments with caffeine and SCH58261 increased the dopamine concentration and TH phosphorylation at Ser31 in the striatum in caffeine- and SCH58261-sensitized mice. Indeed, it has also been reported that 10 mg/kg of caffeine can reverse the catalepsy and decrease the activity produced by DA antagonists in rats [33,34] and has effects on turning in unilateral 6-OHDA-lesioned rodents [35,36]. Caffeine has been found to block the MPTP-induced decrease in the numbers of tyrosine hydroxylase-positive dopaminergic neurons in the striatum in mice [37]. The dosage of 2 mg/kg SCH58261 can significantly improve the ability in an animal model of PD and enhance the therapeutic efficacy of L-DOPA [14]. These observations indicated that in addition to mesolimbic dopaminergic pathway, caffeine in this dosage has effects on the nigrostriatal dopaminergic pathway, and is probably mediated by the adenosine A_2A _receptor. The effect of caffeine and SCH58261 on the neuroadaptation in the striatum, which is the target of mesolimbic and nigrostriatal dopaminergic pathways, may partially explain why they have behavioral sensitization, reinforcing and therapeutic effect in animal models of PD.

Most studies about caffeine and A_2A _antagonists focus on the neuroprotection against dopaminergic neurodegeneration in animal models of PD [38]. In vivo, only two studies showed that chronic treatment with higher doses (25 and 50 mg/kg) of caffeine in rats significantly increased the DA in the striatum, whereas chronic lower dose of caffeine did not alter the DA content [22,39]. Our previous studies showed that lower but not higher doses of caffeine can induce reinforcing and sensitization behavior. To reconcile the apparent discrepancy between the neuroadaptive and behavioral modifications, we chose the lower dosage of caffeine and demonstrated that chronic treatment with lower dose of caffeine (10 mg/kg) can increase the striatal DA in mice. Difference in the animal species and the use of internal standard (2, 3-dihydroxybutyric acid) for recovery of DA in the HPLC quantitation in our study may partially explain the discrepancy.

We also demonstrated that chronic treatment of caffeine and a selective A_2A _antagonist enhance the phosphorylation level of tyrosine hydroxylase at Ser31. Phosphorylation of TH is likely to be of physiological importance in maintaining catecholamine stores because TH is the rate-limiting enzyme in catecholamine biosynthesis and its activity is increased by phosphorylation [40]. TH is phosphrylated at multiple sites. A recent study on intact bovine adrenal chromaffin cells has identified four phosphorylation sites on TH, at Ser8, Ser19, Ser31, and Ser40 [41]. Treatment that increase Ser31 or Ser40 phosphorylation but not the others increase TH activity and catecholamine biosynthesis, and ERK-mediated phosphorylation of Ser31 play a role in dopaminergic related neurological disease [42]. For example, chronic administration of morphine or cocaine increases phosphor-ERK immunoreactivity in the VTA [43], suggesting that dopamine biosynthesis may be elevated in this region. An earlier study has demonstrated that chronic treatment with caffeine (20 and 80 mg/kg for 9 days) increased the tyrosine hydroxylase mRNA levels in both the substantia nigra pars compacta and the ventral tegmental area [23].

In vitro, caffeine at mM concentrations can activate tyrosine hydroxylase in bovine chromaffin cells [44]. Functional striatal hypodopaminergic activity was noted in mice with genetic deletion of adenosine A_2A _receptors [45]. However, genetic deletion of adenosine A_2A _receptors results in persistent rather than transient and intermittent antagonism of the receptor and, in addition, in such study adenosine A_2A _receptors affected basal extracellular dopamine concentration but not total dopamine concentration in striatum. Our findings, together with previous studies, make it plausible that caffeine through adenosine A_2A _receptor-mediated phosphorylation of TH at Ser31, results in the dopaminergic neuroadaptations related to the treatment of PD and mechanism of drug dependence/addiction.

In conclusion, our study demonstrates that low dosages of caffeine and a selective adenosine A_2A _antagonist SCH58261 induce sensitization and cross-sensitization of locomotor activity, which are associated with elevated dopamine concentration and phosphorylation of TH at Ser31 in the striatum. Blockade of adenosine A_2A _receptor may play an important role in the striatal neuroadaptations observed in the caffeine- and SCH58261-sensitized mice.

## Competing interests

The authors declare that they have no competing interests.

## Authors' contributions

CWH and CSW performed animal and pharmacological experiments and the acquisition of data. CWH and THC participated in the experimental conception and design, and were also involved in the interpretation of data, drafting and revising the manuscript.
